# Performance of the SARC-F, SARC-CalF, and calf circumference for sarcopenia case finding in community-dwelling older adults

**DOI:** 10.1007/s41999-024-01060-4

**Published:** 2024-09-19

**Authors:** Hanna Kerminen, Satu Jyväkorpi, Annele Urtamo, Heini Huhtala, Hanna Öhman, Riccardo Calvani, Emanuele Marzetti, Kaisu Pitkälä, Timo Strandberg

**Affiliations:** 1 Faculty of Medicine and Health Technology, The Gerontology Research Center (GEREC), Tampere University, Arvo Ylpön Katu 34, 33520 Tampere, Finland; 2 Department of Geriatrics, Orthopedics and Rheumatology, Università Cattolica del Sacro Cuore, Rome, Italy; 3 The National Nutrition Council of Finland, Helsinki, Finland; 4 Department of General Practice and Primary Health Care, University of Helsinki, Helsinki, Finland; 5 South-Eastern Finland University of Applied Sciences (XAMK), Kouvola, Finland; 6 Faculty of Social Sciences, Tampere University, Tampere, Finland; 7 Department of Geriatric Medicine, University of Helsinki and Helsinki University Hospital, Helsinki, Finland; 8 Fondazione Policlinico Universitario “Agostino Gemelli” IRCCS, L.Go A. Gemelli 8, 00168 Rome, Italy

**Keywords:** Sarcopenia, Screening, Case-finding, Older people, Anthropometry, Sensitivity and specificity, Skeletal muscle

## Abstract

**Purpose:**

We compared the performance of SARC-F, SARC-CalF, calf circumference (CC), and body mass index (BMI)-adjusted CC for sarcopenia case-finding in community-dwelling older adults.

**Methods:**

Data of Finnish participants (women/men *n* = 192/36, mean age (SD) of 76.9 (4.8) years) recruited in the SPRINTT trial (NCT02582138) were used. Sarcopenia was determined as a combination of low muscle function (chair-stand-test) and low appendicular lean mass (ALM) detected by whole-body dual-energy X-ray absorptiometry. Associations of case-finding tools with sarcopenia were analysed using ROC curves and logistic regression.

**Results:**

The rates of probable and confirmed sarcopenia were 95% and 18% in women and 94% and 36% in men, respectively. Performance of CC for sarcopenia (women AUC 0.85 [95% CI 0.78–0.92]/ men 0.85 [95% CI 0.71–1.0]) was superior to that of other tools; (AUC in women/men for SARC-F was 0.57/0.50, for SARC-CalF 0.76/0.79, and for BMI-adjusted CC 0.68/0.66). The best performance was found for a CC cut-off point of ≤ 34 cm in women with sensitivity/specificity 82.4/75.3% and ≤ 36 cm in men with sensitivity/specificity 76.9/87.0%. For each cm decrease in CC, adjusted for age and BMI, there was a 30% increase in the odds of sarcopenia in women (OR 1.30, 95% CI 1.09─1.56). Although there was a similar pattern in men, the results did not reach statistical significance (OR 1.34, 95% CI 0.84- 2.14).

**Conclusions:**

CC was superior to other tools for sarcopenia case-finding. The best performance was found for a CC cut-off point of ≤ 34 cm in women and ≤ 36 cm in men.

## Key summary points

**Aim:**

To compare the performance of different case-finding tools (SARC-F, SARC-CalF, calf circumference (CC), and BMI-adjusted CC) for sarcopenia in community-dwelling older adults.

**Findings:**

CC was the best tool for sarcopenia case-finding. The best performance was found for CC cut-off point of ≤ 34 cm in women and ≤ 36 cm in men.

**Message:**

In clinical practice, measurement of CC is a simple, inexpensive, and reliable tool to identify individuals at risk for sarcopenia for further assessment.

## Introduction

Sarcopenia is an age-related, progressive, and generalised skeletal muscle disorder. It is characterised by low muscle function and low muscle quantity or quality and often leads to poor physical performance [1]. Sarcopenia impairs health-related quality of life and increases the likelihood of several adverse outcomes, such as falls, functional decline, disability, and mortality [1]. The prevalence of sarcopenia in community-dwelling individuals aged ≥ 60 years is approximately 11% in men and 9% in women [2]. In the 65–80 years age group, the prevalence increases by an average of 1.7% every 5 years [3].

Sarcopenia may easily remain undetected in clinical practice unless a systematic case-finding approach is used. The SARC-F has been recommended for sarcopenia case-finding by the European Working Group on Sarcopenia in Older People 2 (EWGSOP2) [4]. It includes five questions that evaluate signs and symptoms related to low muscle function [5]. The revised Asian Consensus Statement on Sarcopenia (AWGS) recommends use of SARC-F, SARC-CalF, or calf circumference (CC) at the community level [6]. SARC-CalF includes the original questions of SARC-F in addition to measurement of CC [7]. CC is an anthropometric measure that correlates with skeletal muscle mass [8–10]. CC comprises not only muscle but also subcutaneous tissue and may therefore overestimate the amount of muscle mass in those with overweight or obesity. To account for this limitation, adjustment of CC for body mass index (BMI-adjusted CC) has been proposed [11].

If case-finding reveals an increased risk for sarcopenia, further testing should be conducted. EWGSOP2 recommends a stepwise approach to diagnose sarcopenia [4]. The first step involves measurement of muscle strength using validated tests such as handgrip strength or chair-stand. If low muscle strength is detected, sarcopenia is probable. The following step is the measurement of lean mass preferably by a whole-body dual energy X-ray absorptiometry (DXA). Appendicular lean mass (ALM), the sum of the lean tissue in the arms and legs derived from a DXA scan, is used as an estimation of muscle mass. A diagnosis of sarcopenia is confirmed if both low muscle strength and low muscle mass are present; the condition is categorized as severe if low physical performance coexists.

As the sensitivity of SARC-F for sarcopenia is low [12, 13], it is not an optimal tool to select patients for diagnostic evaluation as most cases are missed. Although the sensitivity of SARC-CalF is higher than that of the SARC-F [14], almost half of the cases remain undetected. CC has high sensitivity for identifying low muscle mass in middle-aged and older adults [10], older adults with stroke [15], and residents in assisted settings [16]. However, studies on the ability of CC to identify sarcopenia cases in the community are sparse. In particular, more studies performed in Europe are needed. To the best of our knowledge, there are no previous studies on the performance of BMI-adjusted CC for sarcopenia.

In this study, we sought to compare the performance of case-finding tools (SARC-F, SARC-CalF, CC, and BMI-adjusted CC) for sarcopenia in community-dwelling older adults. Considering the high workload and sparse resources in primary health care settings, we aimed to identify case-finding tools that were highly sensitive and at least moderately specific to identify most sarcopenia cases while reliably excluding those without sarcopenia. Furthermore, an ideal tool should be sensitive at identifying early signs of sarcopenia to enable early detection, treatment, and follow-up.

## Methods

### Study design and population

This study was a secondary analysis of the data from Finnish participants that were screened for inclusion in the SPRINTT trial. SPRINTT was a randomised controlled study in 11 European countries with over 1500 participants. It aimed to test the efficacy of multicomponent intervention in the prevention of mobility disability in community-dwelling adults aged ≥ 70 years with physical frailty and sarcopenia [17]. The detailed process for identifying eligible participants is described elsewhere [18]. Most Finnish participants were recruited via letter invitations sent by post. Interested candidates were first phone-screened to identify those with mild challenges in everyday mobility such as slow gait speed, use of cane when walking, or use of handrails when climbing or descending stairs. Suitable participants were invited to the clinical visit where further screening was performed. Finally, eligible participants were randomly assigned to the intervention or a control group. The study protocol was approved by the HUS Regional Committee on Medical Research Ethics in Finland and by the regional ethics committees of all participating institutions. Written informed consent was obtained from all participants that were screened for eligibility, including consent to use information acquired from the screening phase of the trial.

### Clinical assessment

During the screening clinical visit, a structured interview was administered to collect information on medical history, including questions on diseases and perceived symptoms and current medications. In addition, measures of height, weight, and CC and screening tests (SARC-F and Mini Mental State Examination (MMSE) [19]) were performed. Physical performance was assessed using the 400-m walk test [20] and Short Physical Performance Battery (SPPB) that includes an evaluation of standing balance, 4-m walk speed, and the chair-stand test [21]. Whole-body DXA was used to estimate ALM.

### Definition of sarcopenia

For the present study, sarcopenia was defined according to the EWGSOP2 criteria as follows [4]: probable sarcopenia was defined as low muscle strength on the chair-stand test (unable to complete or time to completion exceeding 15 s). Confirmed sarcopenia was defined as the combination of dynapenia and low ALM adjusted by height squared (kg/m^2^). The cut-off points for low ALM were < 5.5 kg/m^2^ and < 7.0 kg/m^2^ for women and men, respectively.

### Sarcopenia case-finding tools

The performance of SARC-F [5], SARC-CalF [7], CC [22], and BMI-adjusted CC [11] for sarcopenia case-finding were evaluated (Table 1).

**Table 1:** Sarcopenia case-finding tools in the community with the recommended cut-off points for suspicion of sarcopenia

	Questions or measurements	Scoring/ adjustment	Cut-off point
SARC-F	How much difficulty do you have in lifting and carrying 10 pounds?	0 None, 1 Some, 2 A lot or unable	≥ 4/10
	How much difficulty do you have walking across a room?	0 None, 1 Some, 2 A lot, use aids, or unable	
	How much difficulty do you have transferring from a chair or bed?	0 None, 1 Some, 2 A lot or unable without help	
	How much difficulty do you have climbing a flight of 10 stairs?	0 None, 1 Some, 2 A lot or unable	
	How many times have you fallen in the past year?	0 None, 1 one to three falls, 2 ≥ 4 falls	
SARC-CalF	SARC-F and measurement of CC	SARC-F score if CC is > 33/34 cm in women/ men, SARC-F score added with 10 points if CC is ≤ 33/ ≤ 34 cm in women/ men	≥ 11/20
CC	Measurement of CC	CC (cm)	≤ 33 cm for women, ≤ 34 cm for men
CC adjusted for BMI	Measurements of CC and BMI	CC value if BMI < 25 kg/m^2^ CC value –3 cm if BMI 25–29.9 kg/m^2^ CC value –7 cm if BMI 30–39.9 kg/m^2^ CC value –12 cm if BMI ≥ 40 kg/m^2^	

*BMI* body mass index, *CC* calf circumference

### Statistical analysis

The characteristics of study participants were described using frequencies and percentages for categorical variables, and median (interquartile range, IQR) or mean (standard deviation, SD) for continuous variables. Predictive capacities of SARC-F, SARC-CalF, CC, and BMI-adjusted CC for sarcopenia were investigated using binary logistic regression analysis, adjusted for age and BMI. The receiver operating characteristic curve (ROC) and the area under the curve (AUC) with 95% confidence intervals (95% CIs) were calculated to clarify the discriminative abilities. For each case-finding tool, sensitivity, specificity, and positive and negative predictive values (PPV and NPV) were calculated using the recommended cut-off points for suspicion of sarcopenia (SARC-F ≥ 4/10, SARC-CalF ≥ 11/20, and CC and BMI-adjusted CC ≤ 33 cm and ≤ 34 cm for women and men, respectively). Additional CC values were tested to identify the best cut-off points for sarcopenia case-finding. A *p* value < 0.05 was considered significant. Data analysis was performed using IBM SPSS Statistics version 29.

## Results

### Baseline characteristics

Characteristics of the study sample are shown in Table 2. Out of 228 participants, 192 were women (84.2%) and 36 were men (15.8%). Median age (IQR) was 76 (80–73) years. Median BMI (IQR) was 27.3 (31.8–23.9) kg/m^2^. SARC-F was normally distributed with a mean score (SD) of 3.5 (1.6). The distribution of SARC-CalF showed two peaks with a median score (IQR) of 4 (11–3). The median CC (IQR) was 36 (38.5–33.0) cm in women and 37.5 (40.4–34.6) cm in men, and the frequency of low CC (women/men ≤ 33/34 cm) was 27% and 22% in women and men, respectively. The median BMI-adjusted CC (IQR) was 33.0 (34.2–31.0) cm in women and 33.4 (34.5–31.6) cm in men, and the prevalence of low BMI-adjusted CC was 59% and 67% in women and men, respectively. Probable sarcopenia was detected in 94.7% of the participants. Sarcopenia was confirmed in 18% of women and in 36% of men (Table 2).

**Table 2:** Baseline characteristics of the study sample according to sex (*n* = 228)

	Women, n = 192		Men, *n* = 36	
	*n*	%	*n*	%
Age, years				
70–74.9	69	35.9	14	38.9
75–79.9	73	38.0	8	22.2
≥ 80	50	26.0	14	38.9
Age, mean (SD)	76.7	(4.4)	78.1	(6.4)
Body mass index, kg/m^2^				
≤ 24.9	63	32.8	8	22.3
25–29.9	66	34.4	15	41.7
≥ 30	63	32.8	13	36.1
Chronic diseases, medications*				
Hypertension	113	58.9	27	75.0
Coronary artery disease	15	7.8	12	33.3
Heart failure	9	4.7	5	13.9
Chronic obstructive pulmonary disease	27	14.1	3	8.3
Previous cancer	30	15.6	9	25.0
Diabetes	31	16.1	10	27.8
Stroke	21	10.9	4	11.1
Falls in the last year	102	53.1	18	50.0
Number of medications				
0–4	98	51	98	50.0
5–9	61	31.8	61	41.7
≥ 10	11	5.7	11	8.3
**Test results**				
MMSE score*, median (IQR)	28	(29–27)	28	(29–26)
SARC-F score				
0–3	98	51.0	23	63.9
≥ 4	94	49.0	13	36.1
SARC-CalF score				
0–10	140	72.9	30	83.3
≥ 11	52	27.1	6	16.7
Gait speed, m/s				
> 0.8	71	37.0	10	27.8
≤ 0.8	121	63.0	26	72.2
SPPB score				
> 8	25	13.0	4	11.1
≤ 8	167	87.0	32	88.9
Calf circumference, cm				
< 35	77	40.1	9	25.0
35–40	78	40.6	16	44.4
> 40	37	19.3	11	30.6
BMI-adjusted calf circumference^1^, cm				
< 30	27	14.1	2	5.6
30–35	134	69.8	26	72.2
> 35	31	16.1	8	22.2
400 m walk				
< 15 min	178	92.7	36	100.0
> 15 min or non-completion	14	7.3	0	0
**Sarcopenia defining criteria**				
*Probable sarcopenia*				
Chair stand -test time > 15 s or non-completion	182	94.8	34	94.4
*Low appendicular lean mass*				
women < 5.5 kg/m^2^, men < 7.0 kg/m^2^	34	17.7	15	41.7
*Confirmed sarcopenia*				
Probable sarcopenia and low appendicular lean mass	34	17.7	13	36.1

*IQR* interquartile range, *MMSE* Mini Mental State Examination, *SD* standard deviation

*Data were missing in 22 women

^1^CC value if BMI < 25 kg/m^2^; CC–3 cm if BMI 25–29.9; CC–7 cm if BMI 30–39.9; CC–12 cm if BMI ≥ 40

### Predictive capacities of case-finding tools for sarcopenia

Univariate analyses showed an association of SARC-CalF, CC, and BMI-adjusted CC with sarcopenia in women while SARC-CalF and CC were associated in men (Table 3). Associations remained significant after adjustment for age and BMI in women. Although there was a similar pattern in men, the results did not reach statistical significance.

**Table 3:** Associations of SARC-F, SARC-CalF, CC, and BMI-adjusted CC for sarcopenia in univariate and multivariable models

	Women, *n* = 192				Men, *n* = 36			
	Univariate		Multivariable*		Univariate		Multivariable*	
	OR	95% CI	OR	95% CI	OR	95% CI	OR	95% CI
SARC-F	1.17	0.91–1.43	1.18	0.90–1.51	1.02	0.68–1.54	1.34	0.75–2.39
SARC-CalF	**1.22**	1.13–1.32	**1.11**	1.02–1.22	**1.47**	1.13–1.91	1.31	0.96–1.80
CC	**1.46**	1.26–1.69	**1.30**	1.09–1.56	**1.53**	1.15–2.04	1.34	0.84–2.14
BMI-adj. CC	**1.22**	1.07–1.40	**1.22**	1.07–1.40	1.34	0.94–1.93	1.33	0.94–1.88

*BMI-adj. CC* calf circumference adjusted by body mass index, *CC* calf circumference, *CI* confidence interval, *OR* odds ratio

*Adjusted for BMI and age, but BMI-adj. CC adjusted only for age

### ROC curves of case-finding tools for sarcopenia

**Fig. 1:**
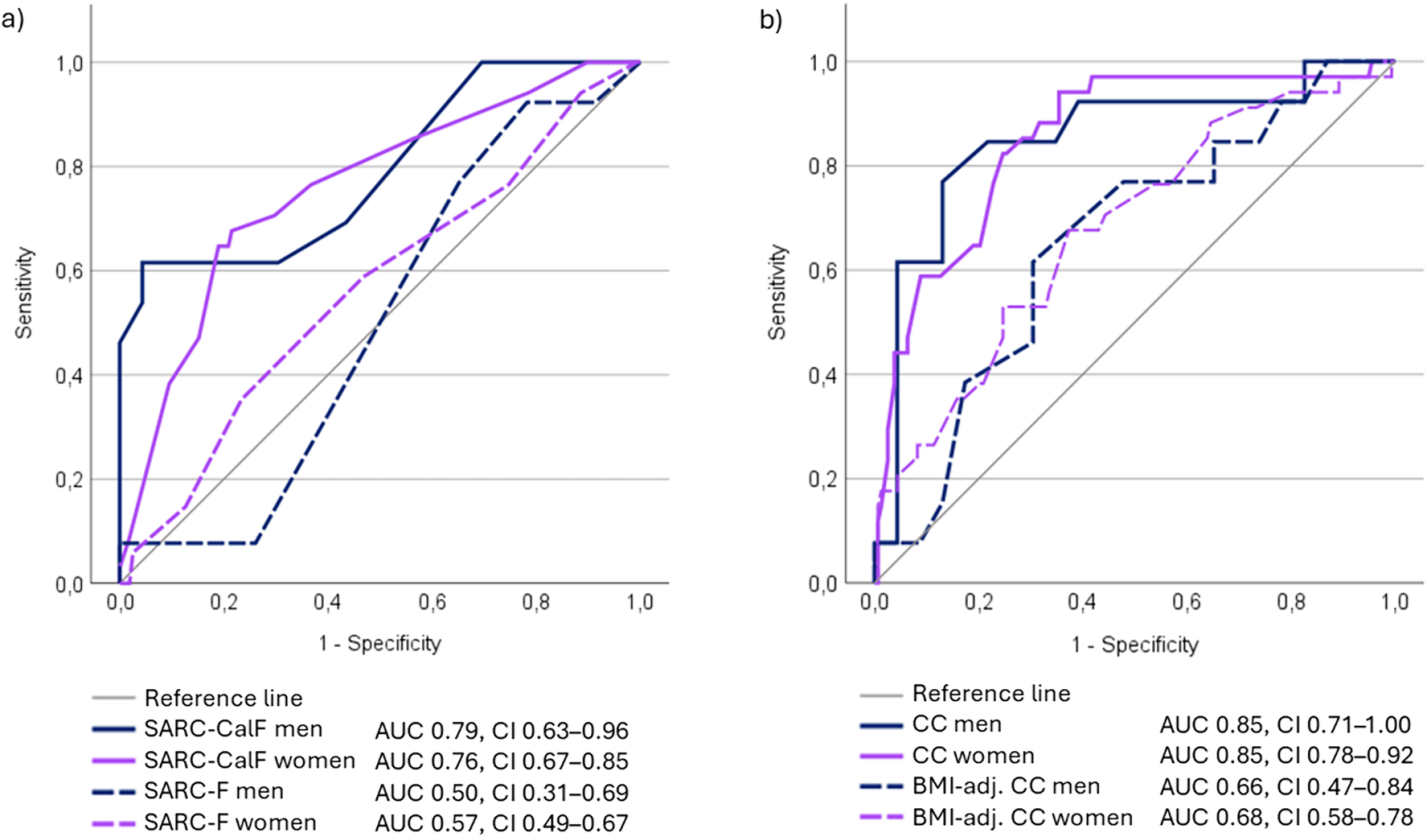
ROC Curves for **a** SARC-F and SARC-CalF, and **b** calf circumference (CC) and CC adjusted by body mass index (BMI-adj. CC) for sarcopenia

CC had the best overall performance for sarcopenia (AUC 0.85 in both sexes) (Fig. 1). The AUCs of SARC-F, SARC-CalF, and BMI-adjusted CC were 0.57, 0.76, and 0.68 in women, respectively. The corresponding values in men were 0.50, 0.79, and 0.66, respectively.

### Sensitivity, specificity, and predictive values

Using the recommend cut-off points, the performance of SARC-F was the lowest; the sensitivity, specificity, PPV, and NPV were 58.8%, 53.2%, 21.3%, and 85.7% in women and 31.8%, 60.9%, 30.8%, and 60.9% in men, respectively (Table 4). SARC-CalF did not provide additional value over CC as the values for CC and SARC-CalF were identical in women and only slightly different in men. Although BMI-adjusted CC had better sensitivity than CC, its specificity and PPV were lower.

**Table 4:** Differential ability of SARC-F, SARC-CalF, CC, and BMI-adjusted CC for sarcopenia case-finding in community-dwelling older adults

	CC cut-off		Women, *n* = 192				Men, *n* = 36			
	point (cm)		Sens	Spec	PPV	NPV	Sens	Spec	PPV	NPV
	W	M	%	%	%	%	%	%	%	%
Usual cut-off points										
SARC-F (≥ 4/10)	–	–	58.8	53.2	21.3	85.7	31.8	60.9	30.8	60.9
SARC-CalF (≥ 11/20)	≤ 33	≤ 34	64.7	81.0	42.3	91.4	46.2	100	100	76.7
CC	≤ 33	≤ 34	64.7	81.0	42.3	91.4	53.8	95.7	87.5	78.6
BMI-adj. CC	≤ 33	≤ 34	76.5	46.2	23.4	90.1	76.9	39.1	41.7	75.0
Additional cut-off points										
CC	≤ 33		64.7	81.0	42.3	91.4	46.2	95.7	85.7	75.9
	≤ 34		82.4	75.3	41.8	95.2	53.8	95.7	87.5	78.6
	≤ 35		88.2	68.4	37.5	96.4	61.5	87.0	72.7	80.0
	≤ 36		94.1	58.8	33.0	97.9	76.9	87.0	76.9	87.0
	≤ 37		97.1	45.6	27.8	98.6	84.6	73.9	64.7	89.5

*BMI* body mass index, *CC* calf circumference, *W* women, *M* men, *NPV* negative predictive value, *PPV* positive predictive value, *Sens* sensitivity, *Spec* specificity

As the CC cut-off point was raised from 33 to 37 cm, there was a constant increase in sensitivity while specificity decreased (Table 4). A CC cut-off point of ≤ 34 cm in women and ≤ 36 cm in men showed the best performance for sarcopenia: sensitivity, specificity, PPV, and NPV were 82.4%, 75.3%, 41.8%, and 95.2% in women and 76.9%, 87.0%, 76.9%, and 87.0% in men, respectively. The performance of SARC-CalF was similar to CC at different CC cut-off points. As the shape of the ROC curve for BMI-adjusted CC was flat, no other cut-off points resulted in better performance.

## Discussion

The findings of this study indicate that, among the various tools considered (SARC-F, SARC-CalF, CC, and BMI-adjusted CC), CC had the best performance at identifying sarcopenia in a well-characterised sample of older adults living in the community.

In previous studies on the prevalence of sarcopenia, considerable heterogeneity has been found depending on the population and diagnostic methods and criteria used [3]. The prevalence of confirmed sarcopenia in our study population was 18% in women and 36% in men, which is higher than previously reported in community-dwelling older adults [2]. This is not unexpected, as the SPRINTT trial targeted older adults with low ALM and physical performance. However, consistent with our results, the prevalence of confirmed sarcopenia was higher in men than in women [2, 3, 23]. The frequency of probable sarcopenia was as high as 94%, which was related to the recruitment method. Surprisingly, the diagnosis of sarcopenia was confirmed in only one fifth of women and one third of men. This finding is consistent with a previous study showing a substantial difference between rates of probable (73%) and confirmed sarcopenia (20%) among hospitalised older men [24]. In contrast, the Korean Frailty and Aging Cohort Study did not find such a large difference between probable (13%) and confirmed sarcopenia (8%) among community-dwelling older adults [23].

In regression models, the SARC-F was not associated with sarcopenia, while the SARC-CalF, CC, and BMI-adjusted CC were associated with sarcopenia in women but not significantly in men. This finding may be explained by the small number of male participants in our study; the point estimates were indeed positive also among men. Each decrease of CC by one cm increased the odds of sarcopenia by 1.3-fold. As previously mentioned, there are studies suggesting an association between low CC and low skeletal muscle mass but only a few studies on associations between CC and sarcopenia. Consistent with our findings, a recent study among hospitalised patients with hip fracture found an association between CC and sarcopenia [25], which was also observed in a Korean study among community-dwelling older adults [9].

The diagnostic performance of SARC-F for sarcopenia in terms of sensitivity and 1-specificity was low; AUC was only about 0.5 and the shape of the ROC curve was flat. Therefore, the sensitivity at the recommended cut-off point (≥ 4/10) was also low and no other cut-off points yielded better sensitivity. In several previous studies, the sensitivity of SARC-F was low but the specificity was high (usually ≥ 80%) [13]. However, in our sample, the specificity of SARC-F was only 53% in women and 61% in men. It is likely that symptom-based queries of SARC-F were not sufficiently accurate for sarcopenia case-finding as, according to the widely used diagnostic criteria for sarcopenia, reduced physical performance is more an outcome rather than a component of sarcopenia [26]. Consequently, questions considering symptoms and outcomes of low muscle function can detect only severe sarcopenia cases.

Our findings suggest that the case-finding tools that include CC have better performance for sarcopenia than SARC-F. This, again, is most probably related to the diagnostic criteria of sarcopenia that considers reduced muscle mass as the main component of sarcopenia, and as such, CC serves as a proxy for muscle mass. Consistent with the present results, previous studies demonstrated that SARC-CalF had better diagnostic performance for sarcopenia than SARC-F [9, 12, 27–29]. Interestingly, SARC-CalF did not add value to CC only; the AUCs of CC were 0.85 in both sexes while the AUCs of SARC-CalF were 0.76 for women and 0.79 for men, with nearly equal sensitivities and specificities at their recommended cut-off points. A possible explanation may be related to the scoring of SARC-CalF that gives equal weight to low CC and all five SARC-F questions. These findings are consistent with a study among hospitalised patients with hip fracture [25] and a Korean study among community-dwelling older adults [9].

According to our findings, it seems that there is no need for BMI-adjustment of CC as a case-finding tool, as CC without adjustment had better performance in sensitivity and 1-specificity. In addition, the specificity of BMI-adjusted CC was lower at the recommended cut-off point than that of CC. This may be at least partly explained by the fact that BMI does not consider fat distribution and therefore CC adjustment by BMI may lead to underestimation of muscle mass in individuals with prevalent abdominal fat accumulation.

In our study, the best performance for sarcopenia was achieved by a CC cut-off point of ≤ 34 cm in women and ≤ 36 cm in men. The optimal cut-off points were greater than in other studies [10, 30]. The recommended CC cut-off points for low muscle mass are based on a large investigation that reported normative CC values of 17 789 community-living adults enrolled in the NHANES study [11]. Low CC was defined as CC values ≤ 33 cm in women and ≤ 34 cm in men. A recent Italian study calculated normative CC values in 11 814 middle-aged and older adults [22]. In individuals aged 70–75 years, the 50th percentile value of CC was 34 cm in women and 36 cm in men. This is consistent with the optimal cut-off points of our study. A few studies have determined the CC cut-off point for sarcopenia in community-dwelling people; these were a Japanese study (cut-off points 33 cm in women and 34 cm in men) and a Korean study (cut-off point 32 cm for both sexes). It is possible that anthropometric characteristics are different between Asian and Western populations [23] and therefore higher CC cut-off points are needed in Western populations to achieve optimal case-finding results.

In a busy clinical practice, measurement of CC is a simple, inexpensive, and reliable tool to identify patients with increased risk for sarcopenia for further assessment and diagnostic evaluation. Considering the high workload of individual general practitioners in the primary care setting, the advantage of CC is that also a trained nurse or a physiotherapist can perform it. Measurement of CC could be routinely performed on certain patient groups at high risk of sarcopenia, for example older adults with frailty or with history of falls, asthenia, or mobility difficulties [31]. This kind of systematic and multiprofessional approach would improve early detection of sarcopenia. Notwithstanding, the questions of SARC-F may be useful for motivating patients for sarcopenia treatment and introducing the subject matter of sarcopenia in clinical practice [4].

A strength of this study is that all tests and measurements were performed by trained personnel among a defined European population. In addition, DXA is considered a reliable method to measure muscle mass. However, it is possible that our results may not be fully representative of community-dwelling older adults, as the target group for the SPRINTT study included older adults with signs of physical frailty and no mobility disability. Thus, our study may overestimate the prevalence of probable and confirmed sarcopenia and show higher SARC-F scores than would be expected in the general population. In particular, PPV and NPV results should be interpreted with caution, as the prevalence of sarcopenia has an impact on these parameters. In addition, the optimal cut-off point for sarcopenia may not be the same in other populations. As the number of male participants in our study was limited, caution is needed when interpreting results from males.

According to EWGSOP2 criteria, hand grip strength and chair-stand test can be used interchangeably to define probable sarcopenia [4]. However, a recent study suggested that these tests define different subgroups of older adults as having probable sarcopenia with significant differences between anthropometric measures [32]. Although both chair-stand test and handgrip strength are associated with CC [33], it is probable that the association of CC with sarcopenia, defined as impaired chair-stand test, is stronger than with sarcopenia defined as impaired hand grip strength. This is an important issue for future research. Further studies are also needed to confirm the findings of the optimal CC cut-off point for sarcopenia case-finding and the usefulness of BMI-adjusted CC as a case-finding tool. As most sarcopenia studies have been performed in Asian and North American populations, more studies are needed among European populations.

In conclusion, the findings of our study suggest that the most accurate case-finding tool for sarcopenia in community-dwelling older adults is measurement of CC. Based on our data, a cut-off point for low CC should be raised to 34 cm in women and 36 cm in men to achieve the best performance. Studies to confirm these findings should be performed in other populations.

## Data Availability

Data are available in anonymous form upon reasonable request addressed to the corresponding author.
